# Thoracolumbar Scoliosis in Pediatric Patients With Loeys-Dietz Syndrome: A Case Series

**DOI:** 10.7759/cureus.36372

**Published:** 2023-03-19

**Authors:** Melissa A LoPresti, Prazwal Athukuri, A. Basit Khan, Marc Prablek, Rajan Patel, Rory Mayer, David F Bauer, Frank T Gerow, Shaine A Morris, Sandi Lam, Vijay Ravindra

**Affiliations:** 1 Department of Pediatric Neurosurgery, Northwestern University Feinberg School of Medicine, Chicago, USA; 2 Department of Neurosurgery, Baylor College of Medicine, Houston, USA; 3 Department of Neurosurgery, Baylor University Medical Center, Dallas, USA; 4 Department of Neurosurgery, Division of Pediatric Neurosurgery, Texas Children’s Hospital, Houston, USA; 5 Department of Pediatric Orthopedic Surgery, Texas Children’s Hospital, Houston, USA; 6 Department of Orthopedic Surgery, Baylor College of Medicine, Houston, USA; 7 Department of Pediatric Cardiology, Texas Children’s Hospital, Houston, USA; 8 Department of Cardiology, Baylor College of Medicine, Houston, USA; 9 Department of Pediatric Neurosurgery, University of California San Diego, San Diego, USA

**Keywords:** connective tissue disorder, scoliosis, pediatrics, management, loeys-dietz syndrome

## Abstract

Background

Loeys-Dietz syndrome (LDS) is a genetic connective tissue disorder that predominantly affects cardiovascular, skeletal, and craniofacial structures. Associated thoracolumbar scoliosis in LDS can be challenging to manage, though other etiologies of pediatric scoliosis have better-defined management guidelines. We examined our institutional experience regarding the treatment of pediatric patients with LDS and scoliosis.

Methodology

In this retrospective study, all patients seen at our pediatric tertiary care center from 2004 through 2018 with a diagnosis of LDS were reviewed, and those with radiographic diagnoses of scoliosis (full-length scoliosis X-rays) were included. Demographic, clinical, and radiographic parameters were collected, and management strategies were reported.

Results

A total of 39 LDS patients whose ages ranged between seven and 13 years were identified. A total of nine patients were radiographically diagnosed with scoliosis, but three patients were excluded due to incomplete medical records, leaving six patients. The median age at scoliosis diagnosis was 11.5 years, with a median follow-up of 51 months. Two patients were successfully managed with observation (average initial Cobb angle (CA): 14°, average final CA: 20.5°). Two were braced, one successfully (initial CA: 15°, final CA: 30°) and one with a progressive disease requiring surgery (initial CA: 40°, final CA: 58°). Of the two who were offered surgical correction, one underwent surgery with a durable correction of spinal deformity (CA: 33° to 19°). One patient underwent a recent correction of aortic root dilatation and was not a candidate for scoliosis surgery.

Conclusions

Principles of adolescent idiopathic scoliosis management such as bracing for CA of 20-50° and surgery for CA of >50° can be applied to LDS patients with good outcomes. This augments our understanding of the treatment algorithm for pediatric patients with LDS.

## Introduction

Loeys-Dietz syndrome (LDS) is an autosomal dominant connective tissue disorder caused by mutations in genes that affect the transforming growth factor beta (TFG-β) pathway, including *TGFBR1*, *TGFBR2*, *SMAD3*, *TGFB2*, and *TGFB3 *[1,2]. Clinically, it is characterized by vascular, skeletal, craniofacial, and cutaneous findings [3,4]. The most common pathological skeletal components include pectus deformity, joint laxity, arachnodactyly, talipes equinovarus, cervical spine deformity, and scoliosis. With 46-62% of patients reportedly exhibiting thoracolumbar (TL) scoliotic deformity [5-7], approximately one-third having structural cervical spine anomalies, and at least half having cervical spine instability [1]. A rare disease, the prevalence of LDS in the overall population is unknown, and the percentage of patients found to have scoliosis is reported to be 62% [5].

Due to the rarity of the disease, there have been limited studies examining the natural history and management of scoliosis in affected individuals. In one of the largest studies, Bessner et al. [5] reviewed 88 cases of pediatric patients with LDS and scoliosis. In this series, 73% of braced patients had progressive disease, with 47% of these patients ultimately undergoing corrective surgery. They reported an increased risk of surgery in LDS patients, including increased blood loss due to underlying vascular fragility. Ultimately, surgery was the definitive treatment for scoliosis in LDS patients in the series [5].

Although there is limited literature focusing on scoliosis in the LDS population, considerable data exist regarding the management of adolescent idiopathic scoliosis (AIS). It is unclear whether similar management principles may be applied to LDS as are applied in AIS. The management of AIS depends largely on the Cobb angle (CA) of the major curve. For curves under 30°, observation with serial radiographs is sufficient; for curves between 30° and 39°, bracing sufficiently reduces the progression of disease; and for curves greater than 50°, surgery is typically recommended, as these curves are likely to progress even beyond skeletal maturity [8,9]. Although this protocol has become the standard in nonsyndromic scoliosis, it has not been studied as a guide in the management of scoliosis in patients with LDS. We aim to describe our experience as a tertiary academic medical center in the treatment of LDS patients with scoliosis. Our experience was largely that of the application of AIS principles to LDS patients. We review the natural history, progression, surveillance, and management options and consider important differences between the LDS population and the nonsyndromic scoliosis population.

## Materials and methods

A retrospective chart review was performed, with Institutional Review Board approval and waiver of informed consent, to identify all cases involving patients with LDS at our institution. Patients were identified from the records of the multidisciplinary program monitoring the cohort of patients with genetically confirmed LDS who were seen from 2004 to 2018. Standard practice for scoliosis screening during this time was the forward bend test for those followed in the multidisciplinary program. If this was abnormal, patients were referred for spine imaging and consultation with either neurosurgery or orthopedic surgery. We included those within this group who were diagnosed with thoracolumbar scoliosis. The diagnosis of thoracolumbar scoliosis was identified from full-length, 36-inch, standing films. Incomplete spinal imaging was considered insufficient to assess scoliosis, and, therefore, patients without complete, 36-inch, standing films were not included. Patients were excluded if no radiographic imaging was available. Demographic data including age and sex; clinical history including presenting symptoms, LDS history, and comorbidities; imaging findings including the below radiographic parameters; functional outcomes including modified Rankin scale (mRS), neurological sequelae of scoliosis, and pain; and genetic testing results were collected by a retrospective review of electronic medical records. In some cases, medical and imaging records were incomplete, and missing data were noted as missing or unable to assess.

Radiographic parameters

Radiographic parameters were assessed using a collection of commonly accepted spinal parameters including the initial major curve angle (CA), current major CA, apical vertebral translation (AVT), coronal global balance (CGB), sagittal vertebral axis (SVA), thoracic kyphosis (TK) and/or lumbar lordosis (LL), and Risser stage (RS). 

**Figure 1:**
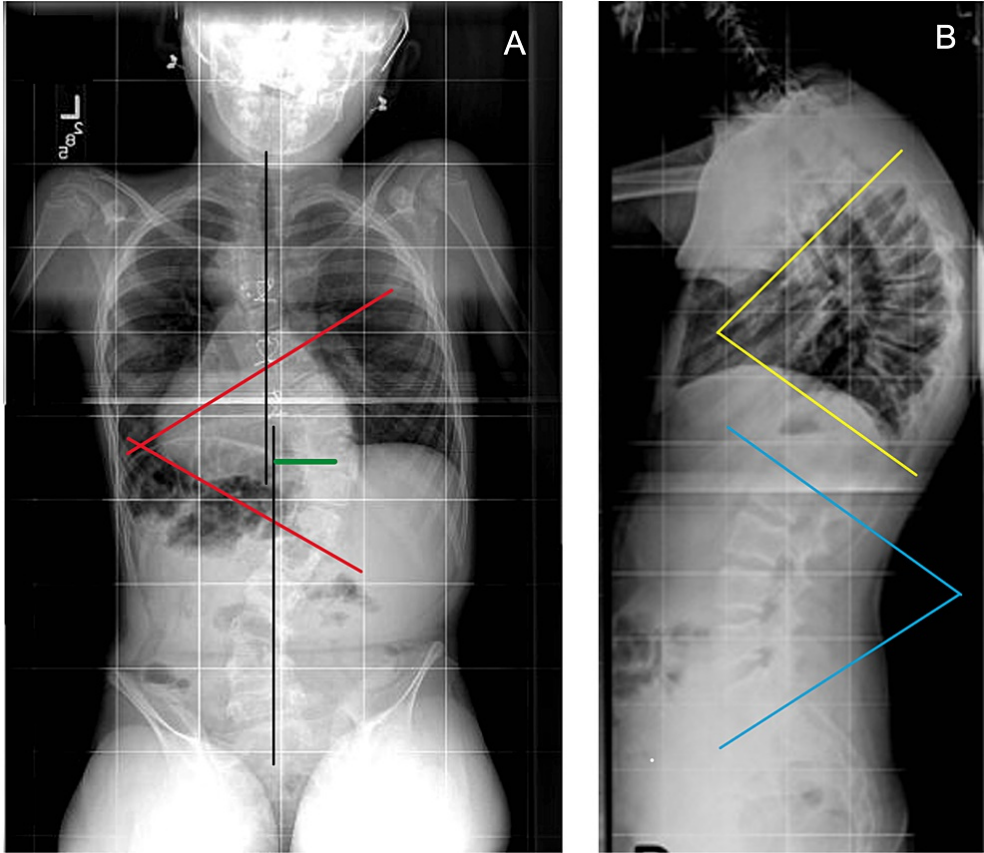
Representative radiographs with measurements. A: Red angle - coronal Cobb angle; upper black line - C7 plumb line; lower black line - central sacral line; green line - apical vertebral translation; distance between black lines - global coronal balance. B: Yellow angle - thoracic kyphosis; blue angle - lumbar lordosis.

The major CA was measured between the superior endplate of the superior-most involved vertebra and the inferior endplate of the inferior-most involved vertebra. The AVT was measured from the midpoint of the apex vertebra of the major CA to the central sacral line. The CGB was measured as the distance from the C7 plumb line to the central sacral line. SVA was measured as the distance from the C7 plumb line to the posterior superior aspect of the S1 superior endplate. TK was measured as the angle from the inferior endplate of T12 to the superior endplate of T4. LL was measured as the angle from the superior endplate of S1 to the superior endplate of L1. These parameters are demonstrated in Figure 1. The Risser stage was obtained from the medical record as recorded by the attending radiologist [10].

## Results

Of the 39 patients with LDS identified at our institution and treated during the study period, nine were diagnosed with scoliosis based on their full-length, 36-inch X-rays. Three of these patients were excluded from our series because of an incomplete medical record. The demographics, characteristics, case descriptions, and management of the six included patients are outlined in Table 1. The median age at diagnosis of scoliosis was 11.5 years (age range = 7-13 years). The follow-up intervals varied by case and are detailed in Table 2 along with radiographic parameters assessed at interval follow-up. The mean and median length of follow-up were 35.2 months and 51 months, respectively (range = 10-67 months).

**Table 1:** Case descriptions of pediatric LDS patients diagnosed with scoliosis. LDS = Loeys-Dietz syndrome; CA = Cobb angle; mRS = modified Rankin scale All DNA variants listed using transcripts SMAD3: NM_005902.3; TGFB2: NM_003238.6; TGFBR1: NM_004612.4; TGFBR2: NM_003242.6 **: Case previously reported in Baskin et al. 2020 [11].

Case number	Gene and variant	Age of scoliosis diagnosis (years)	Sex	Case description and comorbidities	Treatment modality	Initial major curve CA (degrees)	Current major curve CA (degrees)	Time between initial and final imaging (months)	Functional outcomes (mRS)
1	TGFBR1 c.797A>G p.Asp266Gly	7	Female	Asymptomatic at diagnosis of scoliosis. Previously underwent aortic aneurysm repair and experienced bilateral posterior cerebral strokes, but remained ambulatory at baseline	Observation with serial surveillance	14	18	61	0
2	TGFBR2 c.1609C>T p.Arg537Cys	11	Male	Presented with mild back pain at the diagnosis of scoliosis, which was treated medically. Undergoing medical management of aortic root dilation	Observation with serial surveillance	14	23	75	0
3	TGFBR2 Het. (DNA variant not available) p.Arg528His	7	Female	Presented with mild back pain at the diagnosis of scoliosis, which was treated medically. Used wheelchair at baseline because of lower extremity deformities	Observation initially; bracing with progression; surgery offered	40	58	11	4
4	TGFBR2 c.1013C>T p.Thr338Met	13	Male	Asymptomatic at the diagnosis of scoliosis, which preceded the patient being diagnosed with LDS, found incidentally on X-ray that demonstrated an inflexible Schuermann’s thoracic kyphosis	Surgery with thoracolumbar fusion	33	19	41	0
5	TGFB3 Het. 5 Mb deletion of 14q24.3q31.1 incl. TGFB3	12	Male	Asymptomatic at the diagnosis of scoliosis, which was diagnosed before LDS. Experienced mild back pain throughout the course of treatment	Bracing	15	30	67	0
6	SMAD3 Homo. c.532+2T>A**	12	Male	Asymptomatic at the diagnosis of scoliosis. Had progression of major curve with worsening back pain over interval follow-up. However, scoliosis surgery was deferred in favor of treatment for severe aortic root dilation	Surgery offered	48	78	10	0

**Table 2:** Spinal parameters by case CA = Cobb angle; CGB = coronal global balance; SGB = sagittal global balance; AVT = apical vertebral translation; TK = thoracic kyphosis; LL = lumbar lordosis; UTA = unable to assess *: There is a significant discrepancy between this measure and the others for this patient, concerning for an aberrant measure, which may be attributable to imaging type, i.e., flexion/extension films or supine films vs. dedicated standing films. **: Preoperative films. All subsequent imaging is postoperative.

Case number	Imaging interval (months)	CA (degree)	CGB (mm)	SGB (mm)	AVT (mm, absolute value)	TK (degree)	LL (degree)	Risser stage
1	0	14	-5.7	-15	16.8	45	74	0
	7	23	UTA	UTA	25.9	63	73	0
	50	16	-20	-20	18.1	21	61	0
	61	18	-12	-12	15.3	34	56	5
2	0	14	-6.1	-9.7	12.2	3	11	0
	6	20	0	-79.5*	11.4	17	35	0
	21	23	0	-16	35.3	1	1	0
3	0	40	11.1	51.1	33.6	8	4	2
	4	56	27.1	13.3	49.7	15	31	2
	5	36	15.9	42.3	24.0	44	46	2
	11	58	-7.5	32.4	39.9	UTA	UTA	2
4	0**	33	36.3	-10.5	10.5	82	58	UTA
	2	10	21.0	-37.2	0.5	57	51	UTA
	4	14	-0.65	18.3	9.5	42	43	2
	20	22	-3.01	45.6	27.3	58	37	UTA
	41	19	-13.4	19.4	17.6	57	49	5
5	0	15	51.3	-43.8	32.2	22	22	UTA
	6	27	-16.2	19	22	22	22	UTA
	7 (in brace)	16	-31.9	UTA	23.1	UTA	UTA	UTA
	13	27	-9.8	-5.5	26.8	13	65	UTA
	18	27	-30.5	5.5	36.6	12	65	UTA
	25	30	-24.1	UTA	31.9	UTA	UTA	3
	31	30	-16.9	-50.6	24.9	18	57	4
	67	30	12.9	-21.4	17.7	35	68	5
6	0	48	-11.1	50.8	41.5	60	63	UTA
	4	66	-28.4	23.5	53.9	53	66	UTA
	10	78	-0.47	46.2	101.6	85	62	UTA

**Figure 2:**
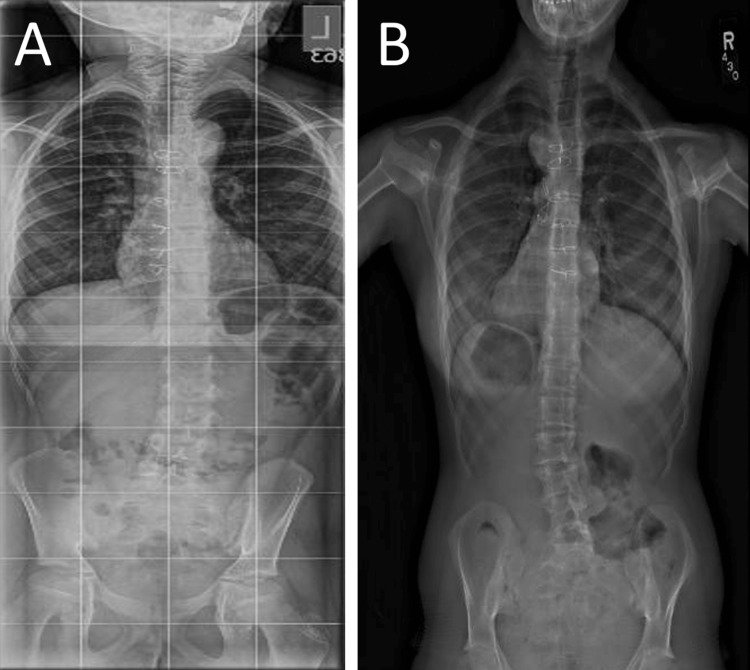
Case one imaging over a five-year period. Initial 36-inch, standing X-ray (A) and most recent X-ray (B) approximately five years apart demonstrating only mild changes to scoliotic pathology. Radiographic imaging obtained over a five-year period demonstrating only mild changes to scoliotic pathology.

Two of six patients were observed (cases one and two). They both had minimal spinal curvatures, with initial major CA <20°. Figure 2 demonstrates the mild imaging changes in case one over a five-year period managed with observation and serial surveillance.

**Figure 3:**
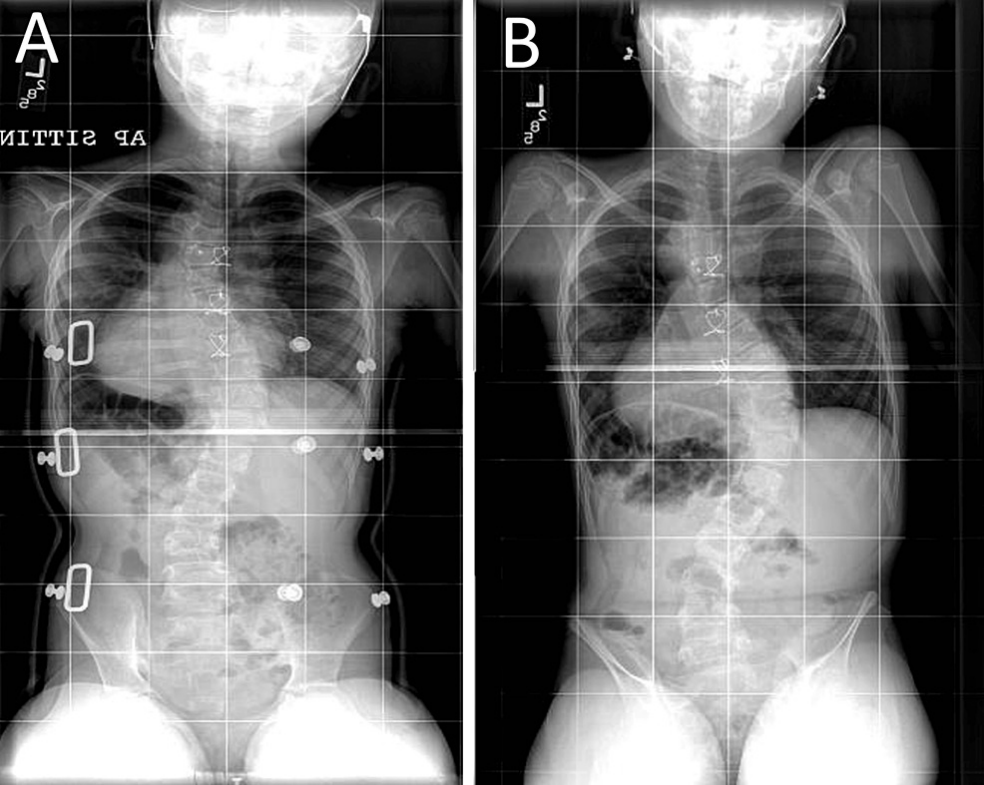
Case three imaging with and without bracing. Imaging with and without bracing. Seated X-rays in A and out B of brace demonstrating progression of the curvature out of brace. Of note, in-brace images were obtained six months before out-of-brace films.

Two of the patients were treated with bracing (cases three and five), although one patient (case three) experienced progression despite treatment with a thoracolumbar spinal orthotic (TLSO) brace. When assessed after one month of bracing, the patient had improved alignment, with a CA of 36° (Figure 3A). However, six months later, when imaged outside of the brace, the patient demonstrated a worsening of the major CA to 58° (Figure 3B). At this point, surgical correction was offered to the patient. The patient transferred care to another hospital in a different city and underwent T3-L4 fusion. Postoperative imaging was not available. Conversely, case five was treated successfully with bracing, having moderate progression of CA from 15° to 30°, which stabilized after achieving Risser stage 4 skeletal maturity, after which bracing was discontinued in favor of observation.

**Figure 4:**
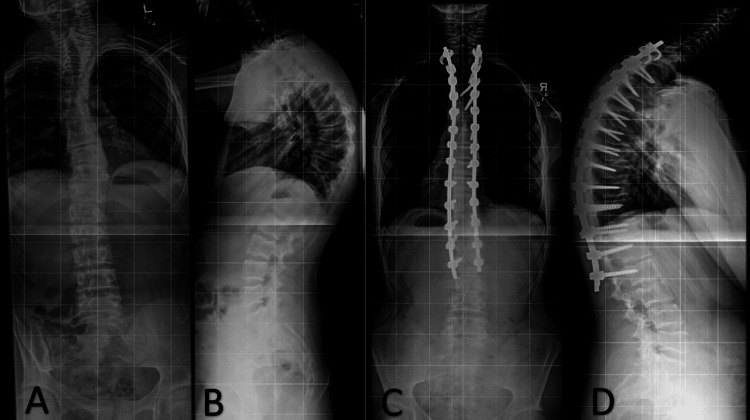
Case four preoperative and postoperative imaging. 36-inch, standing X-ray obtained preoperatively (A - standing posteroanterior X-ray; B - lateral X-ray) and postoperatively (C - standing PA X-ray; D - lateral X-ray). Improved thoracic kyphosis and scoliosis can be seen in images A & B to C & D.

One patient, case four, underwent surgical correction of kyphotic deformity before the diagnosis of LDS. The patient underwent T2-L2 posterior fusion with down-going T2 laminar hooks and T1 and L1 Ponte osteotomies. The estimated blood loss during the procedure was approximately 1,180 mL. The patient did well postoperatively with comparative imaging of preoperative and postoperative films demonstrating improvement in thoracic kyphosis and curvature (Figure 4). Upon follow-up, the patient’s Marfanoid features were identified, and he was later diagnosed with LDS approximately one year after spinal surgery. He subsequently developed spontaneous tension pneumothorax requiring a chest tube and chemical pleurodesis. Since recovering from his pulmonary issues, the patient has progressed well and remained functionally independent. An additional patient (case six) was offered surgery for progressive thoracic curvature and worsening back pain; however, given the patient’s severe aortic root dilatation, he underwent conservative treatment with physical therapy. Scoliosis surgery was postponed and the patient recently underwent aortic root replacement. The patient is not presently a candidate for operative correction of spinal deformity. No patients experienced morbidity related to scoliosis management.

## Discussion

We report our single-institution experience with pediatric LDS patients diagnosed with scoliosis. We implemented various treatment protocols, including conservative observation, bracing, and surgery, based on the unique patient and scoliosis characteristics. In general, our treatment strategy was informed by principles of AIS management. Although we found the responses to treatment were appropriate, there are several key differences to highlight when applying AIS principles to the LDS population.

Skeletal maturity is a key prognostic factor that guides the management of AIS. Skeletal maturity is reported as the Risser stage [12], which extrapolates global skeletal maturity from the degree of ossification in the iliac apophysis on plain X-ray. Patients with lower Risser stages, who are less skeletally mature, are more likely to have progressive worsening of their scoliotic curves over time. One study found that Risser stage 0 patients who were adequately braced based on the current guidelines still had a 41% chance of progressing to surgery [13]. Interestingly, individuals who were braced at Risser stage 0 with open triradiate cartilage were at the highest risk of progressing to surgery, especially if the initial CA exceeded 30° [14]. In our cohort, the treatment paradigm was similar to the management of AIS, with more aggressive surveillance and management of scoliosis in less skeletally mature patients.

One key consideration when treating patients with LDS is the overall health status of the patient. Patients with LDS often have several medical comorbidities, including cardiovascular, cerebrovascular, neurologic, and musculoskeletal conditions, all of which should affect decision-making. Cervical instability is also common in patients with LDS, and may independently require operative fixation. Patient functional status must also be sufficient to participate in postoperative therapies to enhance recovery. Because of the severity of the disease, patients with LDS may self-restrict treatment options. Additionally, patients with LDS present unique surgical risks, including higher rates of reoperation and increased risk of intraoperative bleeding when compared with patients with AIS [5]. Inherent to LDS, an increased risk of blood loss is likely due to intrinsic weakness of blood vessels in patients with LDS, limiting vasoconstriction and self-preservation of blood loss [15-18].

In our case series, conservative treatment with serial surveillance or bracing was opted for in four of six patients, with one patient deferring corrective spine surgery in favor of a needed cardiothoracic procedure. Additionally, the one patient treated surgically had over 1 L of estimated blood loss, which is within the expected range. No patients experienced morbidity related to scoliosis management. We believe the management choices and considerations in this population underscore the importance of special consideration for the overall health and well-being of LDS patients before surgical correction of scoliosis.

Of our patients, two were observed with serial surveillance. Anatomically, they had minor scoliosis (CA <14° at onset). Neither required surgery and both had minimal curve progression. For case number two, there was a significant discrepancy between SGB and the other measurements, concerning for an aberrant measure, which may be attributable to the imaging type used whether it is a flexion or extension film or supine or dedicated standing film. For AIS patients with initial CA of less than 19°, the risk of significant progression of deformity ranges from 2% to 20% depending on the Risser stage [19]. Our experience indicates that scoliotic patients with LDS presenting with mild curves (CA <20°) can be managed with observation, in concordance with data on idiopathic scoliosis.

One patient in our study who had bracing had curve progression from the initial CA of 15° to the final CA of 30°. Bracing was successful because symptoms remained minimal and scoliosis did not progress at the time of skeletal maturity. This contradicts what is described in the literature regarding bracing in LDS. Although bracing is a known effective therapy for AIS patients with moderate disease [9,20], the data for bracing in LDS shows the therapy to be less effective [5]. The disparate response to bracing could be explained by the pathophysiologic difference in idiopathic scoliosis and the syndromic scoliosis of LDS, which is due to global ligamentous laxity. To echo this, scoliotic patients with Marfan syndrome similarly have poor response rates to bracing [21], as lax ligaments inhibit sustained alignment changes from bracing. Although our patient responded well to bracing, this response may represent an outlier of the norm, and, ultimately, more studies are needed to determine the utility of bracing in LDS patients with syndromic scoliosis.

Our study described deformity correction in one case, but surgery was performed before the diagnosis of LDS. Surgery was performed on the diagnosis of Scheuermann’s kyphosis with the purpose of correcting the sagittal deformity. Postoperatively, the patient had sustained resolution of kyphosis, but it is challenging to draw conclusions regarding surgical decision-making from this case. Two patients were recommended surgery, although operative plans are pending. For both, the scoliotic curve was severe and progressive, driving the indication for surgery. Notably, patient six has a rare homozygous mutation of *SMAD3*, causing a severe form of the disease, as previously reported [11]. Posterior fusion achieves coronal correction of 48-67% in AIS patients [22], resulting in improved self-reported measures of self-image, function, and level of activity [23]. Surgery is also effective in correcting scoliosis in patients with Marfan syndrome [24], and in the study by Bressner et al. [5], a curve correction of 69% was reported in the LDS group treated surgically. Based on this, surgical correction may benefit LDS patients with scoliotic curves >50°.

Our single-center experience was largely that of the application of AIS principles to LDS patients. On the whole, the progressive management of scoliotic LDS patients on the grounds of curve severity and progression was effective; however, the scoliotic progression in LDS patients may behave more similarly to that of patients with other types of syndromic scoliosis. In general, syndromic scoliosis is associated with an increased risk of perioperative complications [25]. Similarly, there is a heightened risk of blood loss in neuromuscular scoliosis [26]. Marfan syndrome is perhaps the most comparable to LDS, as both are connective tissue disorders. Almost 60% of Marfan patients have scoliosis, with double and triple curves being more common than in AIS [27,28]. Marfan patients treated surgically have higher rates of dural tears, endure increased blood loss, and require longer fusion constructs [24,29-32]. It may be that patients with LDS undergoing surgical scoliosis correction may need special attention paid to blood loss or require the use of intraoperative adjuncts such as tranexamic acid or cell-saver. Additionally, Marfan patients respond poorly to bracing, with one series of 24 patients reporting a treatment failure rate of 83% [21]. The ineffectiveness of bracing coupled with the higher risk of surgery presents a therapeutic challenge for syndromic scoliosis patients. Ultimately, a study more specific to the management of scoliosis in LDS is required to further elucidate specific treatment algorithms in this group. In sum, we believe that our case series has highlighted the complexities of scoliosis management in the pediatric LDS population.

Limitations

Because of the rarity of LDS in the population, the sample size of our study is small, precluding the assessment of statistical significance from our findings in this cohort. Furthermore, as our study is single-center and retrospective, care was not protocoled or standardized and the data are limited by reporting bias, the quality of the retrospective data from chart review, and practice patterns and preferences within a single-center, limiting the generalizability of our findings. Furthermore, of the patients assessed in this study, a relatively small number of them underwent surgery, which further reinforces the need for larger surgery-focused studies.

Additionally, scoliosis was present in 19% of our patients with LDS, which appears to be lower than reported rates, which are as high as 62% [5,6]. This underestimation may be due to our inclusion requirement of full-length scoliosis X-rays whereas previously published studies included patients diagnosed with scoliosis based on other non-gold-standard imaging modalities such as CT and MRI. Furthermore, given that the extant literature is composed mostly of single-center studies, each reported rate of scoliosis may reflect each center’s patient mix with variation in patient populations and disease severity.

## Conclusions

We demonstrate the radiologic progression of scoliosis in a case series of patients with LDS-associated scoliosis. We explore our management on a case-by-case basis, drawing from our experience and principles of AIS to help guide management in the future. Although the management paradigm for AIS may apply to many patients with LDS, further study is necessary to explore the best management strategies in this unique population.
